# Editorial: Intracranial Atherosclerotic Disease: Epidemiology, Imaging, Treatment and Prognosis

**DOI:** 10.3389/fneur.2021.729377

**Published:** 2021-10-22

**Authors:** Xinyi Leng, Shyam Prabhakaran, Jin Soo Lee, Alex Abou-Chebl, David S. Liebeskind

**Affiliations:** ^1^Department of Medicine and Therapeutics, The Chinese University of Hong Kong, Shatin, Hong Kong, Hong Kong SAR, China; ^2^Department of Neurology, University of Chicago, Chicago, IL, United States; ^3^Department of Neurology, Ajou University School of Medicine, Ajou University Medical Center, Suwon-si, South Korea; ^4^Department of Neurology, Henry Ford Health System, Detroit, MI, United States; ^5^Department of Neurology, Neurovascular Imaging Research Core and UCLA Stroke Center, University of California, Los Angeles, Los Angeles, CA, United States

**Keywords:** stroke, intracranial atherosclerotic disease, imaging, endovascular treatment, stenting

Intracranial atherosclerotic disease (ICAD) is an important cause of ischemic stroke and transient ischemic attack (TIA) worldwide (1). Patients with ischemic stroke attributed to ICAD face a considerable risk of stroke recurrence, despite guideline-recommended treatments, mostly based on findings from the SAMMPRIS (Stenting and Aggressive Medical Management for Preventing Recurrent Stroke in Intracranial Stenosis) trial (2). To date, symptomatic ICAD is treated based on the severity of luminal stenosis in almost all trials and clinical practice, with 70–99% stenoses considered as “high-risk” lesions, despite the fact that nearly half of the recurrent strokes occur in those with “moderate” (50–69%) or even “mild” (<50%) stenosis.

In recent years, advanced imaging techniques have emerged to depict different aspects of ICAD and stroke, providing an extensive amount of data to illustrate the various mechanisms of stroke in ICAD and myriad factors involved in its dynamic evolution and prognosis (3). Yet, research on ICAD still falls far behind that on atherosclerotic disease in other vascular beds, such as coronary and carotid arteries. Numerous questions remain unanswered in this field, e.g., reasons underlying the ethnic difference in ICAD prevalence, evolution in patterns of the disease, differences in risk of stroke relapse and response to treatment in strokes of different mechanisms, differences in acute endovascular treatment (EVT) of large vessel occlusion (LVO) due to ICAD vs. other causes, potential benefit of stenting therapy in selected patients, and safety and efficacy of novel treatment methods.

In this collection, we have included several articles on conventional or novel imaging markers in ICAD and the clinical implications. Yang et al. reported a high prevalence of intracranial arterial calcification in patients with ischemic stroke or TIA, mostly affecting the intracranial portion of internal carotid artery (ICA); they also investigated the association of intracranial arterial calcification, in general or further classified as intimal or medial calcification, with presence of culprit and non-culprit plaques for the index cerebral ischemic event. Among patients with symptomatic or asymptomatic, unilateral, severe stenosis of middle cerebral artery (MCA), Lin et al. evaluated the velocity and extent of cortical venous filling by dynamic CT angiography, and discussed the importance of venous drainage in sustaining cerebral perfusion and affecting clinical outcomes of ICAD patients. In a *post-hoc* analysis of the Chinese IntraCranial AtheroSclerosis (CICAS) Study, Liu et al. found that coexisting ICAD and white matter hyperintensities was associated with an increased risk of unfavorable functional outcome (modified Rankin Scale 3–6) at 1 year, among 2,076 patients with acute ischemic stroke or TIA. With other accumulating studies on ICAD plaque characteristics, collateral circulation and cerebral hemodynamics, etc., these studies reinforced the need for comprehensive assessment of ICAD and coexisting conditions or imaging markers, for more accurate risk stratification of affected patients.

In *post-hoc* analysis of the Mechanisms of Early Recurrence in Intracranial Atherosclerotic Disease (MyRIAD) Study, Sangha et al. revealed a high risk (25%) of early new infarcts (with various manifestations, e.g., borderzone, cortical or territorial infarcts, or in a mixed pattern) within 8 weeks of an initial stroke in symptomatic ICAD patients, indicating the potential value of early imaging follow-up to reveal early, silent ischemic lesions and stroke mechanisms in such patients. In a single-center study, Zhang et al. reported a significantly higher risk of recurrence stroke/TIA in patients with symptomatic, posterior-circulation ICAD than anterior-circulation ICAD (25.5 vs. 14.2%), for which they argued various stroke mechanisms in the two groups as a key explanation, in addition to the differences in the baseline characteristics. These two studies have stressed the need to understand the pathophysiology in ICAD-related stroke and to treat ICAD and the stroke mechanism(s) rather than a single stenosis. This was also emphasized in a Mini Review article in this collection, which reviewed the potential effects of arterial hemodynamics and platelet activity in dominating the response to currently “optimal” medical treatment and outcomes of patients with symptomatic ICAD.

In the past few years, EVT has become a first line treatment in acute ischemic stroke with LVO, after a few successful randomized controlled trials (4). ICAD and embolic occlusion are two main causes of LVO stroke. In a multicenter registry, Lee et al. reported that ICAD as an underlying cause of LVO may not be associated with a higher rate of recanalization failure by mechanical thrombectomy, as compared with LVO with an embolic origin. However, Li et al. found a significantly higher risk of poor functional outcome after EVT associated with ICAD-related LVO than LVO of other causes. Previous studies had also reported conflicting findings (5). On the other hand, acute LVO stroke caused by ICAD or embolic origin may benefit from different EVT strategies. Baek et al. found that rescue endovascular strategy after failure of routine mechanical thrombectomy, preferably in combination with glycoprotein IIb/IIIa inhibitor infusion, may be associated with better imaging and clinical outcomes in ICAD-related LVO, compared with routine mechanical thrombectomy alone. Early identification of the etiology of LVO may help guide treatment strategies: among 164 patients with acute LVO who received EVT, Jin et al. identified the jet-like (pencil-tip-like or line-linked) appearance of contrast filling on the occlusion edge in pre-procedural angiography as an imaging marker of ICAD-related LVO. In addition, Liao et al. have found different pathological compositions of thrombi obtained by mechanical thrombectomy in LVO patients, atrial thrombi obtained during cardiac surgery and carotid plaques obtained by endarterectomy, which may have implications for acute reperfusion therapy in acute ischemic stroke.

Apart from acute EVT, there are several studies on angioplasty and stenting therapy for secondary stroke prevention in symptomatic ICAD patients in this collection. In a multicenter registry study, Guo et al. reported a 8.2% 1-year risk of stroke and vascular death, in patients with symptomatic, high-grade ICAD with imaging evidence of downstream hypoperfusion who received stenting treatment. The risk is comparable with that (8.5%) in the WEAVE (Wingspan Stent System Post Market Surveillance)/WOVEN (Wingspan One-year Vascular Events and Neurologic outcomes) study (6), but much lower than that (over 20%) in the stenting arms of SAMMPRIS (2) and VISSIT (Vitesse Intracranial Stent Study for Ischemic Therapy) trials (7). In a small case series, Hassan et al. reported a zero periprocedural complication rate, and a 7.7% risk of TIA but zero recurrent stroke or death within 6 month, upon using a new generation of drug-eluting balloon-mounted stent in treating medically refractory, symptomatic ICAD (≥70% stenosis). However, symptomatic ICAD in the posterior circulation may bear a higher risk of periprocedural complications, recurrent stroke and death. For instance, Wang et al. reported a high risk (22.7%) of stroke, TIA and death during hospitalization in patients with symptomatic intracranial vertebrobasilar artery stenosis (70–99%) refractory to medical treatment. Luo et al. observed a 55% rate of new cerebral infarctions in diffusion-weighted MR imaging obtained at 72 h after angioplasty and/or stenting treatment of symptomatic basilar artery stenosis, but they did not identify significant associations between plaque characteristics by vessel wall MR imaging and new cerebral infarctions. All these investigations have echoed the rising voice of revisiting the safety and efficacy of stenting therapy (that was overruled as first-line treatment after SAMMPRIS), and associated beneficial and deleterious factors, in selective, symptomatic ICAD patients.

In summary, this collection includes review or original research articles on pathology, pathophysiology, imaging, treatment and prognosis of ICAD and ICAD-related stroke or TIA, with which we intend to elicit further attention on ICAD and more interest in ICAD research around the world. Hopefully in the near future, more accurate risk stratification, and more effective and individualized acute treatment and secondary stroke prevention of symptomatic ICAD will be developed, with the goal “to provide the right treatment, at the right dose or use, for the right patient, at the right time,” as stated in the review article on “precision medicine” for ICAD in this collection.

## Author Contributions

All authors listed have made a substantial, direct and intellectual contribution to the work, and approved it for publication.

## Conflict of Interest

The authors declare that the research was conducted in the absence of any commercial or financial relationships that could be construed as a potential conflict of interest.

## Publisher's Note

All claims expressed in this article are solely those of the authors and do not necessarily represent those of their affiliated organizations, or those of the publisher, the editors and the reviewers. Any product that may be evaluated in this article, or claim that may be made by its manufacturer, is not guaranteed or endorsed by the publisher.
